# Supplementary material for: Tumor-Derived Extracellular Vesicles Inhibit Natural Killer Cell Function in Pancreatic Cancer
Source: Cancers (Basel). 2019 Jun 22;11(6):874. doi: 10.3390/cancers11060874 (PMC6628179; doi:10.3390/cancers11060874)
Supplement: Supplementary file 1 [file cancers-11-00874-s001.pdf]

Supplementary Materials: Tumor-Derived Extracellular Vesicles Inhibit Natural Killer Cell Function in Pancreatic Cancer

Jiangang Zhao, Hans A. Schlößer, Zhefang Wang, Jie Qin, Jiahui Li, Felix Popp, Marie Christine Popp, Hakan Alakus, Seung-Hun Chon, Hinrich P. Hansen, Wolfram F. Neiss, Karl-Walter Jauch, Christiane J. Bruns and Yue Zhao

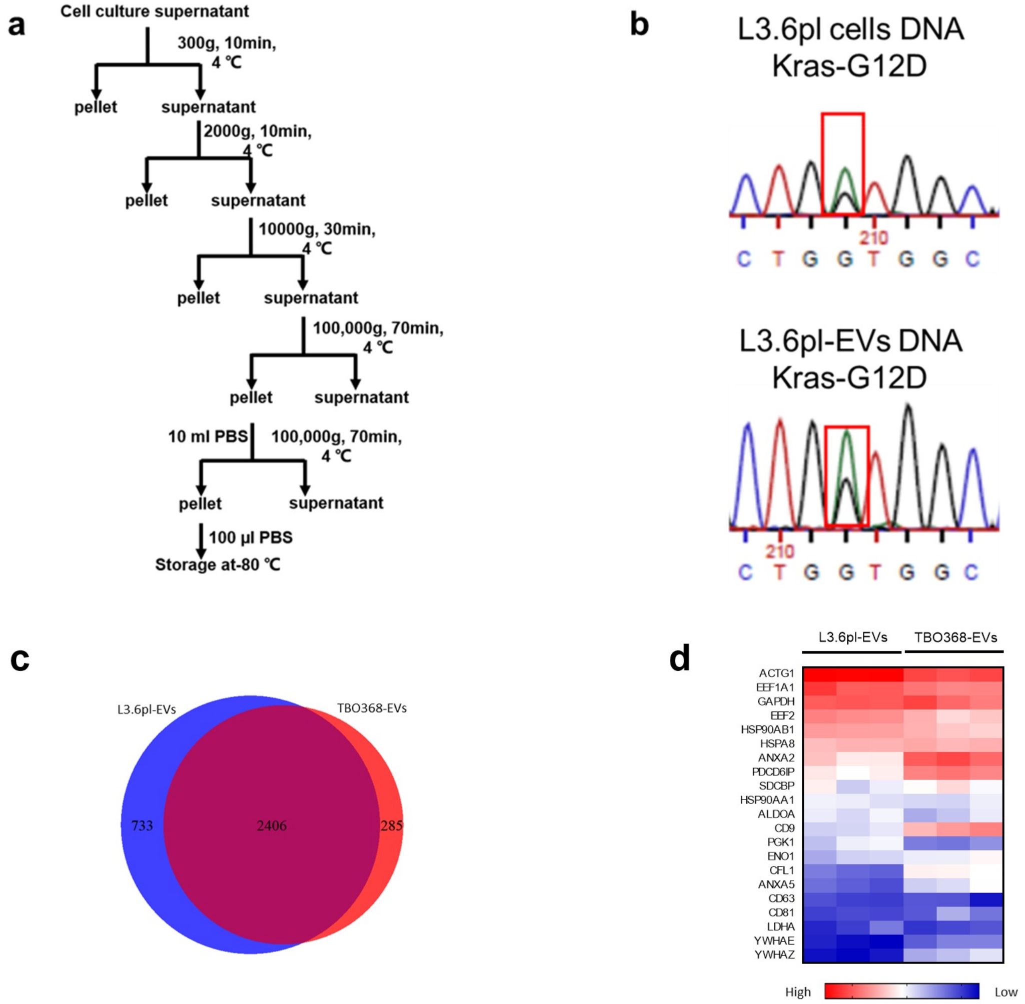

Figure S1. Isolation and analysis of pancreatic cancer-derived EVs. **(a)** EVs were isolated by differential centrifugation and ultracentrifugation. **(b)** Mutant KRAS (G12D) was detected in L3.6pl-derived EVs, which was consistent with that in genomic DNA. **(c)** Proteomic analysis identified 3,139 proteins in L3.6pl-derived EVs and 2,691 proteins in TBO368-derived EVs. The Venn diagram showed an overlap of 2,406 proteins in both samples. **(d)** Heatmap showed enrichment of typical exosomal markers in L3.6pl-derived EVs and TBO368-derived EVs.

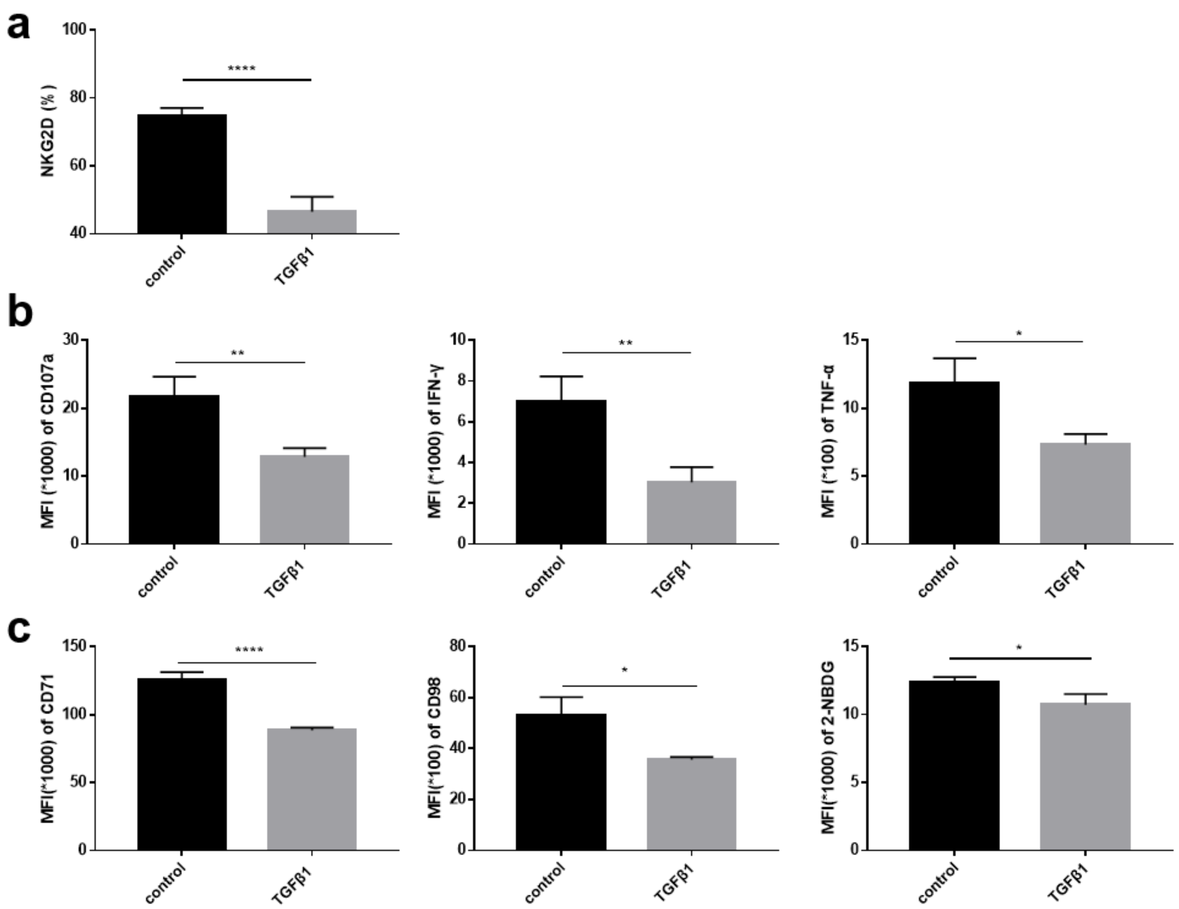

Figure S2. TGF-β1 inhibits NK cell function. **(a)** NK cells were treated with PBS or TGF-β1 for 24 hours. The percentage of NKG2D positive NK cells were analyzed by flow cytometry. **(b)** NK cells pre-treated with PBS or TGF-β1 were co-cultured with L3.6pl cells at a 1:1 ratio for 5 hours. The MFI of CD107a (left), IFN-γ (middle) and TNF-α (right) in NK cells was analyzed by flow cytometry. **(c)** NK cells were treated with PBS or TGF-β1 for 24 hours. NK cells were then analyzed by flow cytometry to determine the MFI of CD71 (left), and CD98 (middle), 2-NBDG incorporation (right).

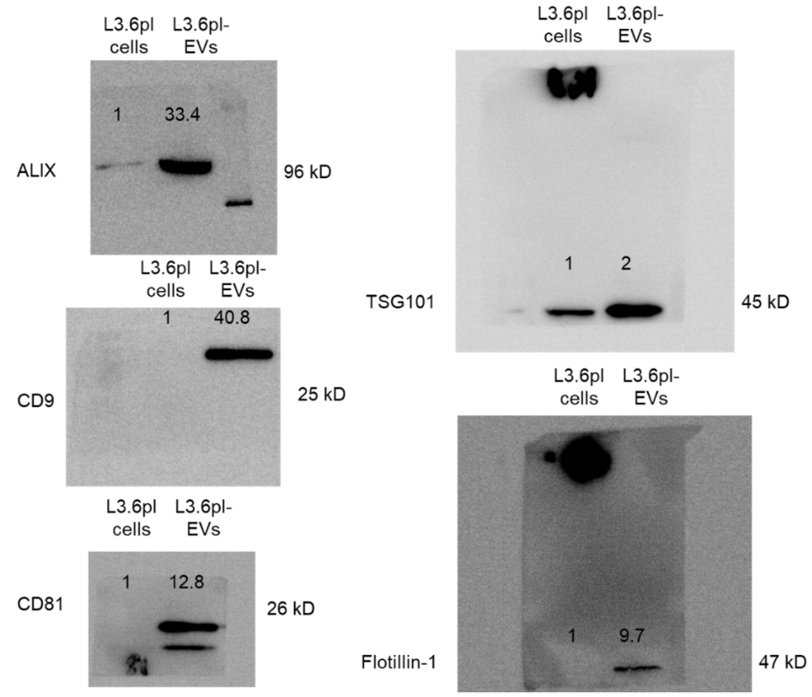

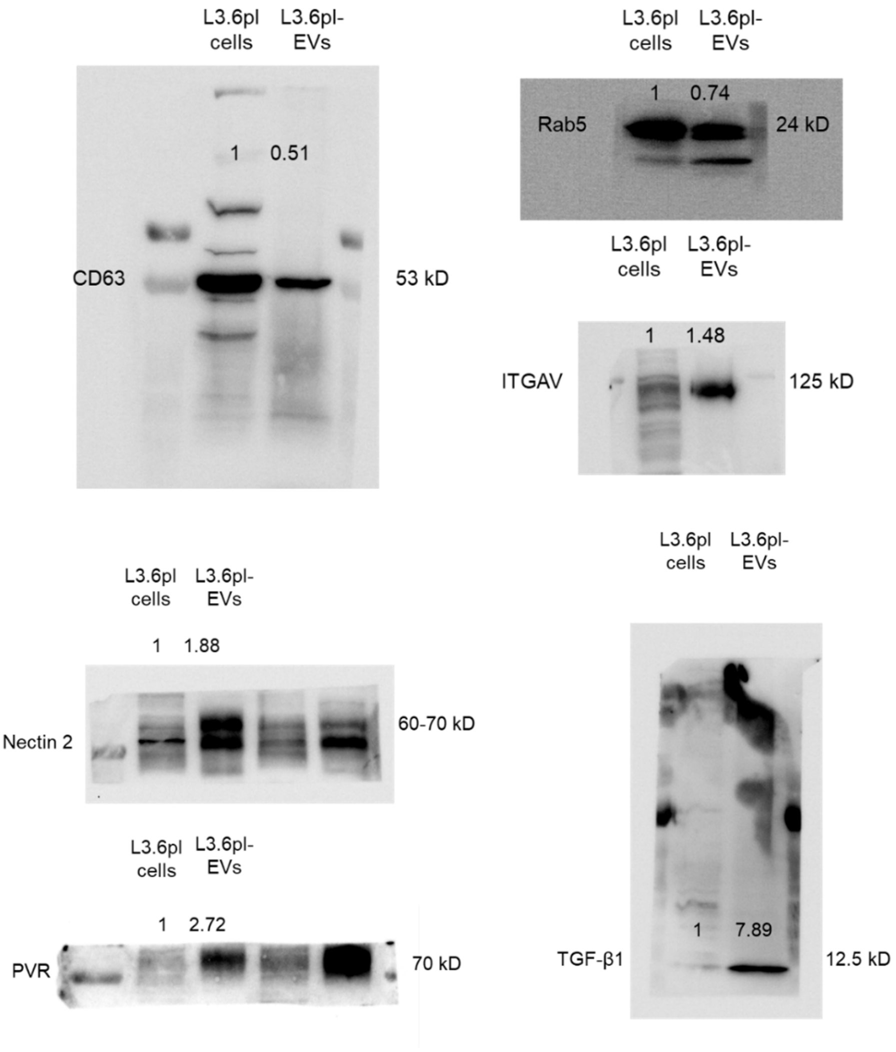

Figure S3. Western blot figures.

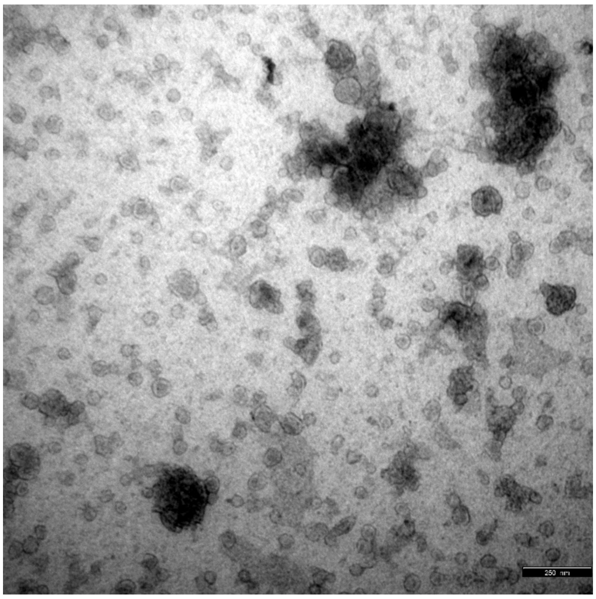

Figure S4. The TEM image of pancreatic cancer-derived EVs.

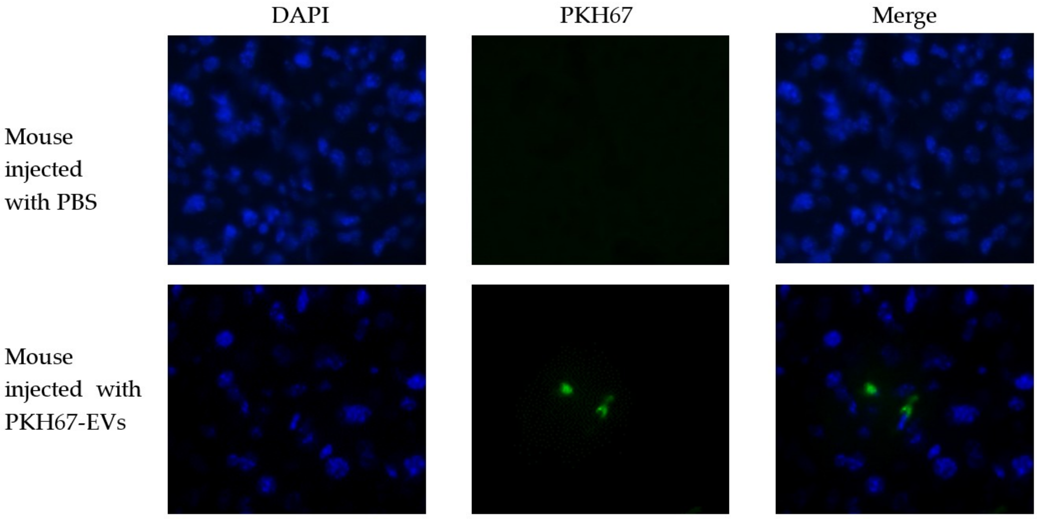

Figure S5. Analysis of liver injected with PBS or PKH67-labeled L3.6pl-derived EVs (green) by confocal microscopy. Nuclei were stained with DAPI (blue). Magnification: 200 ×

Table S1. The clinicopathological characteristics of patients with PDAC.

Clinicopathological Data	Patients (*n* = 30)	Percentage
	n	%
Average age (years)	66.1	
≤60	9	30%
>60	21	70%
Gender		
Male	19	63.3%
Female	11	36.7%
Tumor stage		
T1	4	13.3%
T2	12	40%
T3	11	36.7%
T4	3	10%
Nodal status		
N0	7	23.3%
N1	10	33.3%
N2	13	43.3%
Distant metastasis		
M0	27	90%
M1	3	10%
UICC stage		
I	6	20%
II	11	36.7%
III	10	33.3%
IV	3	10%

Table S2. Antibodies for Western blot.

**Antibody**	**Company**	**Catalog**	**Specificity**	**Host**	**Dilution**
CD9	System Biosciences	EXOAB-KIT-1-SBI	human	rabbit	1/1000
CD63	System Biosciences	EXOAB-KIT-1-SBI	human	rabbit	1/1000
CD81	System Biosciences	EXOAB-KIT-1-SBI	human	rabbit	1/1000
Alix	Santa Cruz	sc-53540	human	mouse	1/1000
TSG101	Santa Cruz	sc-136111	human	mouse	1/1000
Flotillin-1	Santa Cruz	sc-74566	human	mouse	1/1000
Rab5	Santa Cruz	sc-46692	human	mouse	1/1000
TGF-β1	Abcam	ab64715	human	mouse	1/1000
Nectin-2	Proteintech	27171-1-AP	human	rabbit	1/1500
PVR	Proteintech	27486-1-AP	human	rabbit	1/1500
ITGAV	abcam	Ab179475	human	rabbit	1/5000
